# Milk Therapy in Nutritional Disturbances

**Published:** 1915-01

**Authors:** Howard Bucknell

**Affiliations:** 612 Candler Bldg., Atlanta, Ga.


### MILK THERAPY IN NUTRITIONAL DISTURBANCES.

By Howard Bucknell, M. D.

A fruitful reason for lack of recognition of the therapeutic value of various methods of substitute infant feeding is the fact that many children appear to thrive, and in fact, do thrive, on carelessly prescribed milk mixtures and proprietary foods.

As in other fields of therapeutics we must consider prophylactic and remedial measures.

In feeding healthy children the problem is, to provide material for the best growth and development and, to prevent disease.

The problem, in feeding children who are ill, is to cure the morbid condition, prevent loss of strength and tissue, interfere as little, and for as short a time as possible, with normal development, and to sustain life, during illness, by allowing the less impaired functions to continue to carry on vital processes while the more impaired, or exhausted, functions are rested.

The advantage of therapeutic feeding is that while nourishing the infant and supplying material for growth and development, definite changes may be made to occur in the digestive tract by the use of the food alone, and that, when necessary, drugs may be used with increased effect.

The cause of scurvey and the ease with which the condition can be prevented or cured by dietetic measures has long been generally recognized.

Rachitis can, to a large extent be prevented, and certainly can be greatly benefitted by prescribing proper food.

For the infant, sick or well, the one best food during the early months of life is good breast milk.

According to the teachings of Czerney, Finkelstein, Heubner and Myer, the adjective good is superfluous, as they assert in unison that all mother's milk is always good for all infants, and that when a baby does not gain and thrive on the breast it is due to: (I) Faulty technic, (II) Wrong quantity, (III) Disturbances affecting the mother secondarily.

Finkelstein states that the contra-indications to breast-feeding are: Tuberculosis, a few severe general diseases, some acute diseases when the mother is very sick, and pregnancy. He states that syphilis in the mother of the child is not a contra-indication, nor is Angina, Diphtheria, Pneumonia, or Typhoid, unless the mother is very ill. He also states that most drugs do not go into the milk, and that those which do are excreted in such small amount they do no harm.

However this may be in German Hospitals, we frequently see well children become sick children, while nursing healthy mothers, because of an improper proportion of the ingredients of the breast milk for that particular child.

Frequently a milk too high in cream causes disturbances that continue in spite of control of quantity and time of feeding, till an added food changes the proportion of cream to the other food principals used; while, less frequently, the milk being ample in quantity it is too low in caloric value to give material for proper development. Still these difficulties may frequently be overcome by treating the mother, and when this is insufficient it is seldom necessary to do more than give auxiliary feedings while letting the child have the benefit of the mother's milk. These benefits are actual, though probably not entirely satisfactorily explained and certainly in many cases exert a definite healing effect in intestinal disturbances and probably transmit for a considerable period, an immunity, acquired by the mother, to the nursing infant.

An important factor much stressed by the German pediatrists is the dithesis or congenital idiosyncracy of the child.

Another, which is greatly neglected by writers on this subject, is geography. A diet ideal for Eskimos would be absolutely impossible for infants in the tropics.

An extremely valuable fact concretely expressed by Grule is that there are three definite amounts of food for the infant; the smallest amount on which it can live, the best amount for normal development, and the largest amount, which it can take without causing disturbance.

In considering quantity of food, caloric feeding is of undoubted value as a guide. Without caloric calculation, the danger is always present of starvation or of gross overfeeding with the grave possibility, in cases where tolerance is impaired, of a fatal paradox reaction.

In calculating the number of calories permissible, the condition of the functions of the digestive tract must of course be carefully considered during a nutritional disturbance, and, when convalescence occurs, the calculations must include consideration of the birth-weight, and the weight that should normally have been acquired had the infant developed properly. Like all guides, caloric feeding should be used as a safeguard and followed with due consideration of the conditions present and not as an infallible routine.

For example: if enough fat is ingested to supply 500 calories and 300 calories of fat are excreted in the stool, it is obvious that the entire number of calories are not being used.

So one frequently sees an infant kept on a food containing sufficient caloric value for steady gain with a stationary weight in spite of an apparently normal stool for a considerable period, and then there will be a steady gain with no change in the feeding formula. In these cases it would seem reasonable to suppose that the function of digestion and metabolism for one or more of the ingredients in the formula had become more active and that food which had been wasted was being used.

There is a difference between non-digestion and indigestion as we use the term.

A valuable aid to a better understanding of the nutrition of the infant is to consider the function of digestion and metabolism of each food principle as a separate entity, and to realize that while the function of digestion and absorption of fats may be greatly impaired, the function of digestion and absorption of carbohydrates may be much less impaired, and that the less impaired functions may use food supplied to maintain life while the exhausted function is resting.

This same may be true of each of the digestive functions.

Let us use a simile. If the left arm is able to lift a 5 lb. weight a certain number of times without exhaustion, the right arm 10 lb., the left leg 10 lb., and the right leg 15 lb., and each continued to do the work it was able to do, with periods of rest, there would normally develop an increase in power, but if the left arm was made to do the work of the right, it would become exhausted and incapable of lifting any weight whatever. Except for the general exhaustion of the body as a whole, the weight-lifting power of the right arm and of both legs would be unimpaired, and by diminishing the total amount of work to be done, they would be able to continue to do service until the exhausted left arm would gradually regain its weight-lifting function. And so with the digestive functions. As no two children have exactly the same weight-lifting ability for each arm and leg, so no two have exactly the same ability to use to advantage exactly the same quantity and proportion of fats, proteids, carbohydrates and salts. Serious failures are bound to occur when one feeds John on the amount of sugar on which Mary thrived just because Mary thrived. If one must run the risk of mistake it is usually better to increase too slowly than to over-tax functions and have the terrifying sudden loss of weight that seems inexplicable from the appearance of the stools.

Czerney, Heubner, Finkelstein and their associate workers have given extremely valuable assistance in therapeutic feeding by showing the role of proteids, carbohydrates, fats and milk salts, alone, and in relation to each other in producing definite changes in the digestive tract, and the body as a whole.

Until very recently it has been taught that dilution of cow's milk was necessary for infant feeding because of its too high proteid content, but Finkelstein teaches that is necessary because of the large amount of whey salts.

The German school shows that proteid in excess causes the stools to become more alkaline, allaying intestinal irritation, delaying peristalsis, and promoting constipation, while Loeb has shown that calcium salts have a similar effect, and that the combined casein and calcium have a distinctly constipating effect.

That lactose in excess causes, by bacterial action, the formation of acid stools, thus irritating the intestine, and increasing peristalsis leading to diarrhoea.

That whey, in which potassium and sodium salts predominate, increases peristalsis and that calcium salts in the casein retard peristalsis.

That fats increase either diarrhoea or constipation, depending on which condition is present.

The Czerney and Finkelstein followers have heated arguments as to how the milk salts accomplish their physiological effects; one trying to prove that the formation of soluble irritant soaps by the combination of sodium and potassium salts with fats present are responsible for the diarrheal condition, and that the formation of insoluble non-irritant calcium soap stools is the reason for constipation, while the other asserts that the action of the salts alone is sufficient explanation, but as to the essential facts both agree.

In many intestinal conditions cereal water is undoubtedly of great value. A common mistake is to continue this treatment too long and starve the patient. It should be considered as a temporary expedient. When proper food is again resumed it is necessary to know the lowest life-sustaining amount that can be used in order that too large an amount shall not be given, thereby causing a relapse with increased difficulties or a fatal termination.

It is a frequent pastime for many to condemn unqualifiedly the Finkelstein Eiweiss-Milch, and its therapeutic value in diarrheal conditions. Usually those who enjoy this pastime do not prepare it properly, nor use Finkelstein's methods to make diagnosis of conditions to be treated by the Finkelstein technic.

Others as readily condemn the Kellner Malt-Soup idea because they have used it in cases where it was distinctly contra-indicated as a therapeutic measure. If using a definite technic of therapeutic feeding as routine, it is necessary to make diagnosis of the condition to be treated according to the methods of the originator of the technic.

At the Evelina Hospital, Dr. Mann gets most excellent results with whole milk in which the proteid is rendered more completely available, in diarrheal cases, without changing the proportion of fats or sugar. At first glance this appears to be directly at variance with the German technic, but by rendering proteid completely available it has the effect of increasing the proportions of proteid, while the quantity is actually unchanged.

Routine stool examinations should never be neglected. One must determine if the stool is alkaline, or acid, if it is fermentative, foul, contains fat or casein curds, or mucus. It is also important to know if there is undigested starch, and if the excreted fat is in the form of soaps, fatty acids or neutral fats. In testing the stool for starch it is well to remember that many dusting powders are made of this substance.

The German idea in increasing the proteid percentage in diarrheal conditions is not only to get a casein and calcium effect, but at the same time to allow a greater amount of the other food principles to be used by keeping the casein and calcium in excess.

It would seem a mistake to follow any technic too closely as circumstances modify the use of every technic.

An accurate understanding of principles is of the greatest possible value. When knowledge of principles and experience in use of these is gained the actual technic can be modified as opportunity offers. If one method only is used the idea will become fixed that it is the only possible means of accomplishing the desired end, while it is only a single adaptation of an infinite number of ingenious modifications.

There is no universal food that can be used to feed every adult without regard to his symptoms. There is no universal prescription that can be given to infants just because they happen to be infants without regard to their condition or symptoms, and there is no universal formula on which an infant can be properly fed just because it happens to have lived a definite number of weeks. It is wrong to use a food that produces unfavorable symptoms, and try to control those symptoms with drugs.

No one would condone the thoughtless jumping from one drug to another whose physiological actions were unknown or unconsidered in the hope that perhaps one might prove useful, and that the others might not injure, nor should the physician use food ingredients thoughtlessly or carelessly or without careful study of the needs of the case and the therapeutic value and physiological action of the food principles used.

612 Candler Bldg., Atlanta, Ga.

